# Cardiac Effects of Antiretroviral-Naïve versus Antiretroviral-Exposed HIV Infection in Children

**DOI:** 10.1371/journal.pone.0146753

**Published:** 2016-01-20

**Authors:** Nikmah S. Idris, Michael M. H. Cheung, Diederick E. Grobbee, David Burgner, Nia Kurniati, Cuno S. P. M. Uiterwaal

**Affiliations:** 1 Department of Child Health, Faculty of Medicine University of Indonesia - Cipto Mangunkusumo National General Hospital, Jakarta, Indonesia; 2 Julius Global Health, Julius Centre for Health Sciences and Primary Care, the University Medical Centre Utrecht, Utrecht, Netherlands; 3 Department of Pediatrics, University of Melbourne; Heart Research Group, Murdoch Children's Research Institute, Royal Children's Hospital, Parkville, Melbourne, Australia; London School of Hygiene and Tropical Medicine, UNITED KINGDOM

## Abstract

**Background:**

Cardiac involvement in HIV infected children has been frequently reported, but whether this is due to HIV infection itself or to antiretroviral treatment (ART) is unknown.

**Methods:**

This cross sectional study involved 114 vertically-acquired HIV-infected (56 ART-naive, 58 ART-exposed) and 51 healthy children in Jakarta, Indonesia. Echocardiography was performed to measure dimensions of the left ventricle (LV) and systolic functions. We applied general linear modeling to evaluate the associations between HIV infection/treatment status and cardiac parameters with further adjustment for potential confounders or explanatory variables. Findings are presented as (adjusted) mean differences between each of the two HIV groups and healthy children, with 95% confidence intervals and p values.

**Results:**

Compared to healthy children, ART-naïve HIV-infected children did not show significant differences in age-and-height adjusted cardiac dimensions apart from larger LV internal diameter (difference 2.0 mm, 95%CI 0.2 to 3.7), whereas ART exposed HIV infection showed thicker LV posterior walls (difference = 1.1 mm, 95%CI 0.5 to 1.6), larger LV internal diameter (difference = 1.7 mm, 95%CI 0.2 to 3.2) and higher LV mass (difference = 14.0 g, 7.4 to 20.5). With respect to systolic function, reduced LV ejection fraction was seen in both ART-naïve HIV infected (adjusted difference = -6.7%, -11.4 to -2.0) and, to a lesser extent, in ART-exposed HIV infected children (difference = -4.5%, -8.5 to -0.4). Inflammation level seemed to be involved in most associations in ART-exposed HIV-infected, but few, if any, for decreased function in the ART-naive ones, whereas lower hemoglobin appeared to partially mediate chamber dilation in both groups and reduced function, mainly in ART-exposed children.

**Conclusions:**

ART-naive HIV infected children have a substantial decrease in cardiac systolic function, whereas the ART-exposed have thicker ventricular walls with larger internal diameter and higher mass, but less functional impairment.

## Background

HIV infection in children remains an important global health challenge, not only because the incidence is still rising in some parts of the world, [1] but also due to its systemic and long term consequences. Cardiac involvement is one of HIV infection complications that raises much attention as it may manifest early with fatal consequences in childhood or remain clinically silent until adulthood with cumulative exposure to additional risk factors.[2]

Reports about cardiac abnormalities in HIV-infected children have come from both the pre- and post- highly-active antiretroviral treatment (ART) era, when studies mainly differed in the type of study population, whether focusing on untreated or treated subjects.[3] Despite being frequently reported, the exact mechanisms underlying cardiac pathology in HIV-infected children are not clearly understood, including the differentiation of HIV infection effects from treatment. Such knowledge may be obtained by studying measurements in the pre- and post- highly active ART era, but historical comparisons usually leave comparability concerns. Also, studies on the effects of ART on the cardiovascular system rarely involved populations that had not been exposed to any single ART at all, [4] making effect differentiation difficult. Concerns may also arise as some studies reported unadjusted findings, [5] while using convenience samples as references that despite clinical indications, had no anomalies on echocardiography. Furthermore, although the ART introduction has resulted in a dramatic decrease in overt cardiac complications, such as cardiomyopathy, subclinical structural and functional changes may still exist despite treatment.[6] It remains unclear whether this arises from HIV infection itself or due to the complex interplay between HIV infection and ART. Such delineation would only be possible by making direct comparisons of cardiac effects between ART-naïve or ART-exposed HIV infected and healthy children. Here, we investigated the effects of vertically acquired ART-naïve and ART-exposed HIV infection on childhood cardiac structures and functions through comparisons with healthy children.

## Material and Methods

### Study design and population

We performed a cross-sectional analysis on 114 HIV infected and 51 healthy children enrolled in an ongoing cohort established in June 2013 in Jakarta, Indonesia. HIV infected children (56 ART-naïve and 58 ART-exposed) were recruited from the pediatric HIV clinics of Cipto Mangunkusumo National General Hospital, Koja District Hospital, and the *Indonesian Planned Parenthood Association* (IPPA), Jakarta Indonesia. The ART-naïve HIV infected group included 49 children who had never received or were within the first week of antiretroviral therapy (ART) initiation and 7 children who had been off treatment for at least 5 years. Among ART-treated children, two had just started treatment in our clinic after previous management in another hospital with potential compliance issues. We excluded children with conditions suggestive of non-vertically acquired HIV (HIV-negative parents and previous blood transfusion).

Healthy children with the same age range were invited specifically for this research, from the area around the Cipto Mangunkusumo hospital by directly approaching parents and community leaders and providing leaflets explaining the purpose and procedures of the study. Of the total of 55 children invited, 3 declined to participate because parents/guardians were not able to accompany the child to the hospital or refusal to blood sampling and 1 was excluded due to dysmorphic features suggestive of a syndrome.

Given this study was a part of a larger study aiming at investigating cardiovascular effects of HIV infection in children, in which carotid intima media thickness (cIMT) was the primary outcome, sample size was estimated as such to achieve an 80% power to detect a difference of 25 micrometers in cIMT between the HIV and healthy children with a level of significance of 0.05 using a two-sided two-sample t-test and an estimated standard deviation of 40. This resulted in a minimum of 42 children to be recruited to each group.

For every child participant, written informed consent was obtained from parents or guardians on behalf of children. Children also gave assent and we did not perform echocardiography and blood test for those who refused. This study has been approved by the Ethics Committee of Faculty of Medicine University of Indonesia, Jakarta, Indonesia.

### Measurement of exposure

HIV infection diagnosis was established based on the WHO 2007 criteria. Under 18 months of age, diagnosis was established based on a positive HIV DNA or RNA PCR examination, whereas for those aged 18 months or older, it was defined by positive HIV antibodies. Disease severity was classified based on the WHO clinical and immunological staging criteria [7] applied at the time of echocardiography measurement. The CD4 cell level to define immunological staging was obtained from routine clinical data within 3 months of enrolment. Viral load examination was not routinely done in our HIV care setting due to financial constraints and only selectively performed as indicated by treating physicians.

### Echocardiography measurement

To measure cardiac structural and systolic function parameters, we performed standard (B-mode, M mode) echocardiography, following the recommendation of the American Society of Echocardiography.[8] Children were assessed in supine position using a Phillips Sonos 4500 machine with 7 and 12 MHz phase array transducers and simultaneous reference ECG recordings. Using M-mode, the following cardiac dimensions were measured at parasternal long axis view: left ventricular posterior wall thickness at end-diastole (LVPWd), internal diameter at end-diastole (LVIDd), and mass (LV mass); the same approach was used to measure LV fractional shortening (FS) as a systolic function parameter, reflecting degree of LV diameter shortening between end-diastole and end-systole. Relative wall thickness (RWT) was calculated as LVPWd divided by ^1^/_2_ LVIDd.[9] Other functional parameters sought were LV ejection fraction (LVEF, the fraction of blood volume pumped out of the LV at each heartbeat, calculated as the ratio of stroke volume over LV end-diastolic volume measured using the Simpson biplane method), and tricuspid annular plane systolic excursion (TAPSE), indicating right ventricular systolic function. All parameters were measured in 3 to 4 cardiac cycles and the averages were taken.

Echocardiograms were available for 155 children (50 ART-naïve and, 54 ART-exposed HIV infected, 51 healthy). The remaining children did not have echocardiography data because they refused the examination (2 subjects), were transferred to another hospital (1), died (1), or did not show up for the appointment (6). Examinations were performed by one of the investigators (NSI) for the first 134 children, while the remaining children were examined by two trained cardiology fellows. The agreement between examiners were priorly assessed in 10 children, revealing an intraclass correlation coefficient (two-way mixed modeling) of 86.7 (95%CI 79.0 to 91.7). Results were also randomly validated offline on stored digital loops by a pediatric cardiologist consultant (MMD). All procedures were done in the Echocardiography Laboratory of Department of Child Health University of Indonesia—Cipto Mangunkusumo National General Hospital, Jakarta, Indonesia.

### Potential confounders and intermediary variables

Age and height were a priori set as potential confounders in the association between HIV infection and cardiac parameters as there variables are likely to be associated with HIV infection and heart dimension and function. [10] Height as an indicator of nutritional status, may also act as a mediator as HIV infection likely affects nutritional status, by which the heart might be impaired. As inflammation, assessed by hsCRP level, [11] blood pressure, or hemoglobin level may mediate the effect of ART-naive or ART-exposed HIV infection on heart structures and functions, we also explored their possible intermediary roles.

We obtained data on age and smoking exposure using a standard questionnaire filled by parents or caregivers, whereas body weight and height were measured using a standard method. [12] Laboratory analyses for hemoglobin and hsCRP levels were performed at Cipto Mangunkusumo Clinical Pathology Laboratory, Jakarta; personnel were blinded to HIV infection/treatment status and other clinical data.

### Data analysis

Baseline characteristics are described as mean, median, or proportion as appropriate and differences across HIV status groups were tested using Chi-square, oneway ANOVA, or Kruskal-Wallis tests, respectively.

To investigate the effects of HIV infection on cardiac structures/functions, we performed univariable and multivariable general linear modeling with HIV status as a fixed factor and cardiac parameters (LVPWd, LVIDd, LV mass, RWT, EF, FS, TAPSE) as separate dependent variables. The same models were used for adjustment for age and height as potential confounders. We considered height a better indicator of growth and body size than weight as it is less affected by acute changes, such as volume status or other pathological conditions (ascites, organ enlargement) that may accompany HIV infection in children. To describe the potential explanatory roles of hsCRP level, systolic and diastolic blood pressures, and hemoglobin level, we graphically presented effects of adding each of these to the confounder adjusted model. We also did sensitivity analyses by only including children who had never received ART at all and by excluding 2 ART-exposed children previously managed in another hospital.

Findings are presented as crude and adjusted mean differences with 95% confidence intervals and corresponding p-values. A 95% confidence interval not including zero, corresponding to a p-value of <0.05 was considered statistically significant. All analyses were performed using SPSS version 19 and RStudio 0.98.507 for Mac.

## Results

Table 1 shows baseline characteristics of study subjects. Children with HIV infection, both ART-naïve or -exposed, came from lower socioeconomic status. Family history of CVD was more frequent in HIV-infected subjects, particularly in the ART-naïve, compared to healthy children. ART-naïve HIV-infected patients were younger and had smaller body size among others. Compared to ART-exposed HIV-infected children, ART-naïve children had more comorbidity, were in more severe immunodeficiency states, and had the lowest mean hemoglobin levels among others. The median duration of treatment in ART-exposed group was 2.2 (0.1–9.9) years and only 5 children had a regimen containing PI drugs.

**Table 1:** Baseline characteristics of study subjects.

Characteristics	ART-naïve HIV n = 50	ART-exposed HIV n = 54	Non-HIV N = 51	P-value
Age (years)*, median (range)	3.7 (0.6–11.5)	5.3 (0.6–12.2)	6.4 (2.4–14.0)	0.02
Male gender, n (%)	23 (46.0)	25 (46.3)	24 (47.1)	0.99
Low SES, n (%)	20 (40.8)	31 (57.4)	10 (19.6)	0.001
Parental smoking exposure after birth, n (%)				
Mother	4 (8.5)	6 (11.3)	0 (0)	0.06
Father/other household member	37 (74.0)	47 (87.0)	34 (66.7)	0.04
Parental smoking exposure during pregnancy, n (%)				
Mother	7 (15.2)	8 (15.4)	1 (2.0)	0.04
Father/other household member	37 (74.0)	45 (83.3)	35 (68.6)	0.21
Parent(s) as primary caregiver, n (%)	27 (54.0)	17 (31.5)	42 (84.0)	<0.001
Spontaneous delivery, n (%)	37 (78.7)	45 (83.3)	39 (79.6)	0.82
Ever breastfed, n (%)	34 (73.9)	40 (75.5)	50 (98.0)	0.002
Family history of CVD	16 (34.0)	14 (26.4)	3 (6.1)	0.003
Body weight (kg), n = 147	13.0 (6.8)	17.4 (6.1)	22.5 (9.5)	<0.001
Body height (cm), n = 147	93.4 (19.1)	104.8 (16.9)	116.5 (17.7)	<0.001
Body mass index (kg/m2), n = 147	14.3 (2.3)	15.4 (2.4)	15.9 (2.8)	0.001
Waist circumference (cm), n = 126	48.8 (9.8)	51.1 (6.5)	55.1 (8.5)	0.002
Waist-to-hip ratio	1.02 (0.87–1.20)	1.03 (0.83–1.16)	0.98 (0.88–1.09)	0.001
Systolic BP (mmHg), n = 136	101.0 (10.9)	99.9 (10.5)	102.3 (9.6)	0.50
Diastolic BP (mmHg), n = 136	62.3 (9.6)	60.4 (7.7)	61.1 (7.4)	0.55
Hb (mg/dL) level, n = 142	11.2 (6.9–19.8)	11.5 (4.2–14.6)	12.7 (10.2–14.6)	<0.001
hs-CRP, median (range), nmol/L	37 (0–2759)	48 (371–5507)	5 (0–155)	<0.001#
Chronic infection (ever)*	43 (91.5)	31 (64.6)	0 (0)	<0.001
Severe infection* n = 131	20 (51.3)	5 (12.2)	0 (0)	<0.001
HIV clinical stage 3 or 4	48 (90.6)	32 (61.6)		<0.001
CD4 absolute level (n = 74)	441 (2–2716)	1255 (4–2301)	-	<0.001
CD4% (n = 71)	12 (0–49)	30 (4–47)	-	<0.001
Viral load (n = 23)	320 (59–47914)	544 (0–1.2*10^6^)		
LVPWd (mm)	5.8 (1.4)	6.7 (1.6)	6.0 (1.4)	**
LVIDd (mm)	31.0 (6.3)	32.7 (4.9)	33.4 (4.1)	**
IVSd (mm)	6.5 (1.4)	7.1 (1.4)	6.8 (1.4)	**
LV mass (gram)	43.2 (18.6)	58.5 (20.3)	50.1 (17.2)	**
LV mass index (gram/m2)	74.2 (26.7)	84.1 (25.5)	62.3 (12.9)	<0.001
RWT	0.38 (0.12)	0.42 (0.12)	0.36 (0.09)	**
LVEF (%)	61.3 (13.2)	63.1 (9.7)	67.7 (5.4)	**
LVFS (%)	33.4 (13.2)	33.5 (9.7)	36.0 (5.4)	**
TAPSE (mm)	16.5 (3.5)	18.9 (4.2)	20.2 (3.3)	**

Note: LVPWd = left ventricular posterior wall thickness at end-diastole; LVIDd = left ventricular internal diameter at end-diastole; IVSd = inter ventricular septal thickness; RWT = relative wall thickness; LV mass = left ventricular mass; LVEF = left ventricular ejection fraction; LVFS = left ventricular fractional shortening; TAPSE = tricuspid annular plane systolic excursion.

^#^Kruskal Wallis test; Values are means (SD) unless otherwise indicated.

*Chronic infection included tuberculosis, persistent diarrhea, and chronic otitis media; severe infection included sepsis, pyelonephritis, pneumonia, and CNS infection.

**See Tables 2 and 3

**Fig 1:**
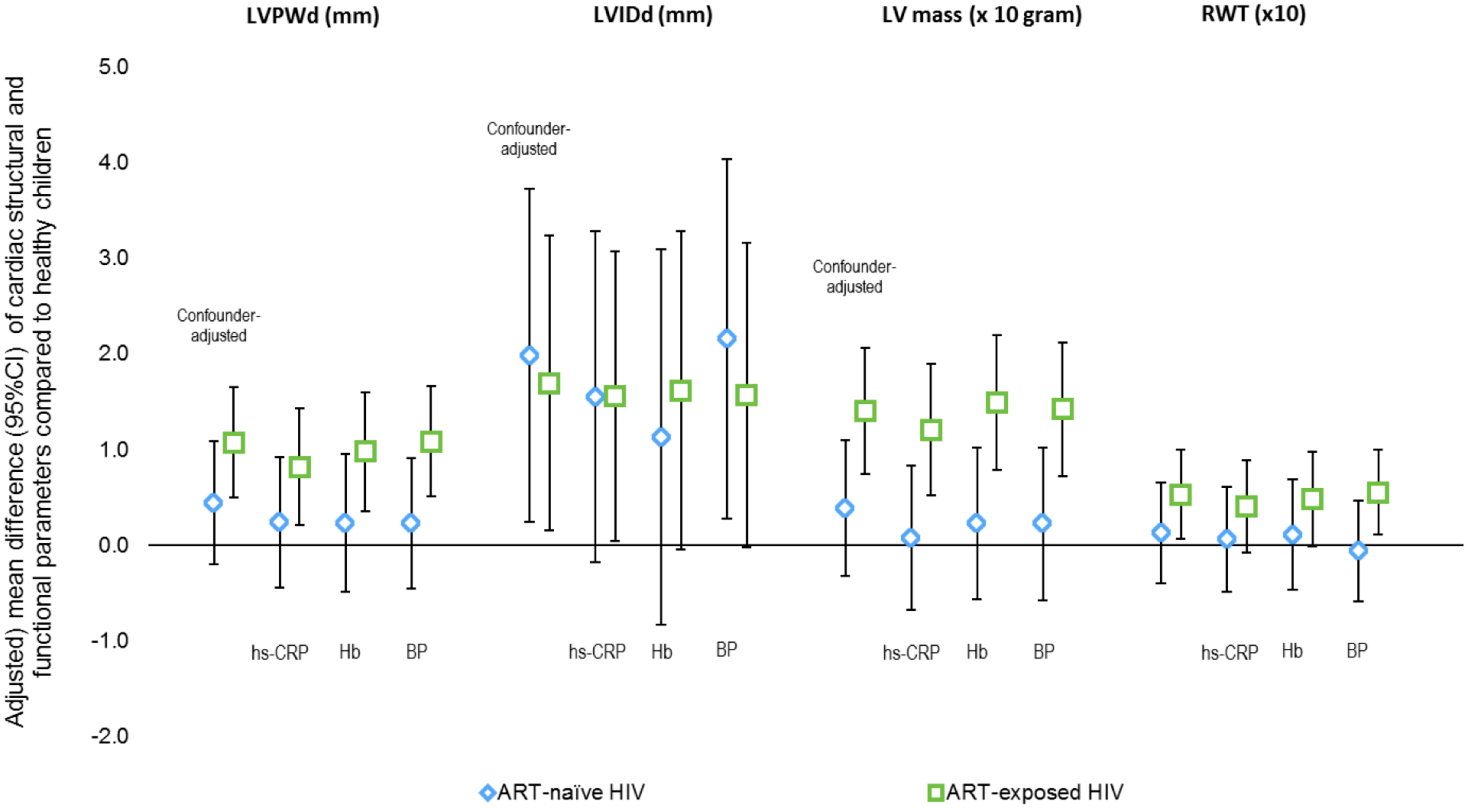
Influence of potential explanatory variables on cardiac structural estimates in ART-naive and ART-exposed HIV infected children. Note: LVPWd = left ventricular posterior wall thickness at end-diastole; LVIDd = left ventricular internal diameter at end-diastole; LV mass = left ventricular mass; RWT = relative wall thickness; Hb = hemoglobin level; BP = systolic and diastolic blood pressure.

Table 2 shows effects of ART-naïve and ART-exposed HIV infection on cardiac structures. While ART-naïve HIV-infected children tended to have smaller LV mass (difference -7.0g, p = 0.09), the difference disappeared after adjustment for age and height. ART-naïve HIV-children seemed to have smaller LVIDd (difference -2.5mm, p = 0.02), but after being adjusted for age and height, they actually had larger LVIDd than healthy children (difference = 2.0mm, p = 0.03). This larger LVIDd appeared to be explained by inflammation (Fig 1) and hemoglobin levels. There was no difference in posterior wall thickness in end-diastolic (LVPWd) between ART-naïve HIV infected and healthy children (Table 2).

**Table 2:** Effects of HIV infection on cardiac structures.

		Crude				Model 1			Model 2		
			Mean difference	95%CI	P	Mean difference	95%CI	P	Mean difference	95%CI	P
LVPWd (mm)	Non-HIV		Ref			Ref					
	ART-naïve HIV	All 50	-0.25	-0.83 to 0.33	0.40	0.14	-0.44 to 0.74	0.63	0.43	-0.21 to 1.08	0.18
		41 Never ART	-0.34	-0.93 to 0.25	0.26	0.13	-0.50 to 0.75	0.69	0.44	-0.23 to 1.12	0.20
	ART-exposed HIV		0.67	0.10 to 1.24	0.02	0.86	0.30 to 1.41	<0.001	1.07	0.50 to 1.65	<0.001
IVSd (mm)	Non-HIV		Ref			Ref			Ref		
	ART-naïve HIV	All 50	-0.23	-0.82 to 0.34	0.42	0.11	-0.48 to 0.70	0.73	0.27	-0.37 to 0.91	0.42
		41 Never ART	-0.31	-0.89 to 0.27	0.30	0.10	-0.53 to 0.70	0.78	0.28	-0.39 to 0.95	0.41
	ART-exposed HIV		0.28	-0.29 to 0.86	0.33	0.46	-0.01 to 1.02	0.11	0.45	-0.13 to 0.10	0.13
LVIDd (mm)	Non-HIV		Ref			Ref			Ref		
	ART-naïve HIV	All 50	-2.45	-4.53 to -0.37	0.02	0.21	-1.53 to 1.94	0.82	1.98	0.24 to 3.72	0.03
		41 never ART	-3.11	-5.26 to -0.96	0.005	0.11	-1.77 to 2.0	0.91	2.08	0.17 to 0.40	0.03
	ART-exposed HIV		-0.70	-2.71 to 1.31	0.50	0.35	-1.25 to 1.95	0.67	1.69	0.15 to 3.23	0.03
RWT	Non-HIV		Ref			Ref			Ref		
	ART-naïve HIV	All 50	0.03	-0.02 to 0.07	0.24	0.02	-0.03 to 0.06	0.52	0.01	-0.04 to 0.06	0.64
		41 never ART	0.02	-0.03 to 0.07	0.44	0.01	-0.04 to 0.06	0.74	0.004	-0.06 to 0.06	0.90
	ART-exposed HIV		0.06	0.02 to 0.10	0.007	0.06	0.01 to 0.10	0.01	0.05	0.06 to 0.10	0.03
LV mass (gram)	Non-HIV		Ref			Ref			Ref		
	ART-naïve HIV	All 50	-6.96	-14.98 to 1.05	0.09	0.41	-6.62 to 7.43	0.91	3.83	-3.28 to 10.93	0.29
		41 never ART	-8.19	-16.32 to -0.05	0.049	2.46	-4.46 to 9.37	0.48	6.86	0.09 to 13.64	0.047
	ART-exposed HIV		8.39	0.42 to 16.36	0.04	11.88	5.12 to 18.65	<0.001	13.99	7.44 to 20.54	<0.001

Note: Model 1: adjusted for age; Model 2: adjusted for age, and height

ART exposed HIV infected children had thicker LV posterior walls (LVPWd), both in crude (0.7mm, p = 0.02) and after adjustment for age and height (1.1mm, p <0.001), and higher LV mass (crude 8.4g, p = 0.04; adjusted 14.0g, p<0.001) although the difference in inter ventricular septal thickness (IVSd) was not statistically significant. The RWT also tended to be higher in ART-exposed HIV infected children both on crude (difference 0.06, p = 0.007) and age-and-height adjusted analysis (difference = 0.05, p = 0.03). On adjusted analysis, ART exposed HIV infected children also had larger LVIDd (1.7mm, p = 0.03). Inflammation level seemed to partially explain the effects of ART-exposed HIV infection in children on the observed structural changes, while hemoglobin level appeared to account for larger internal diameter only (Fig 1).

**Fig 2:**
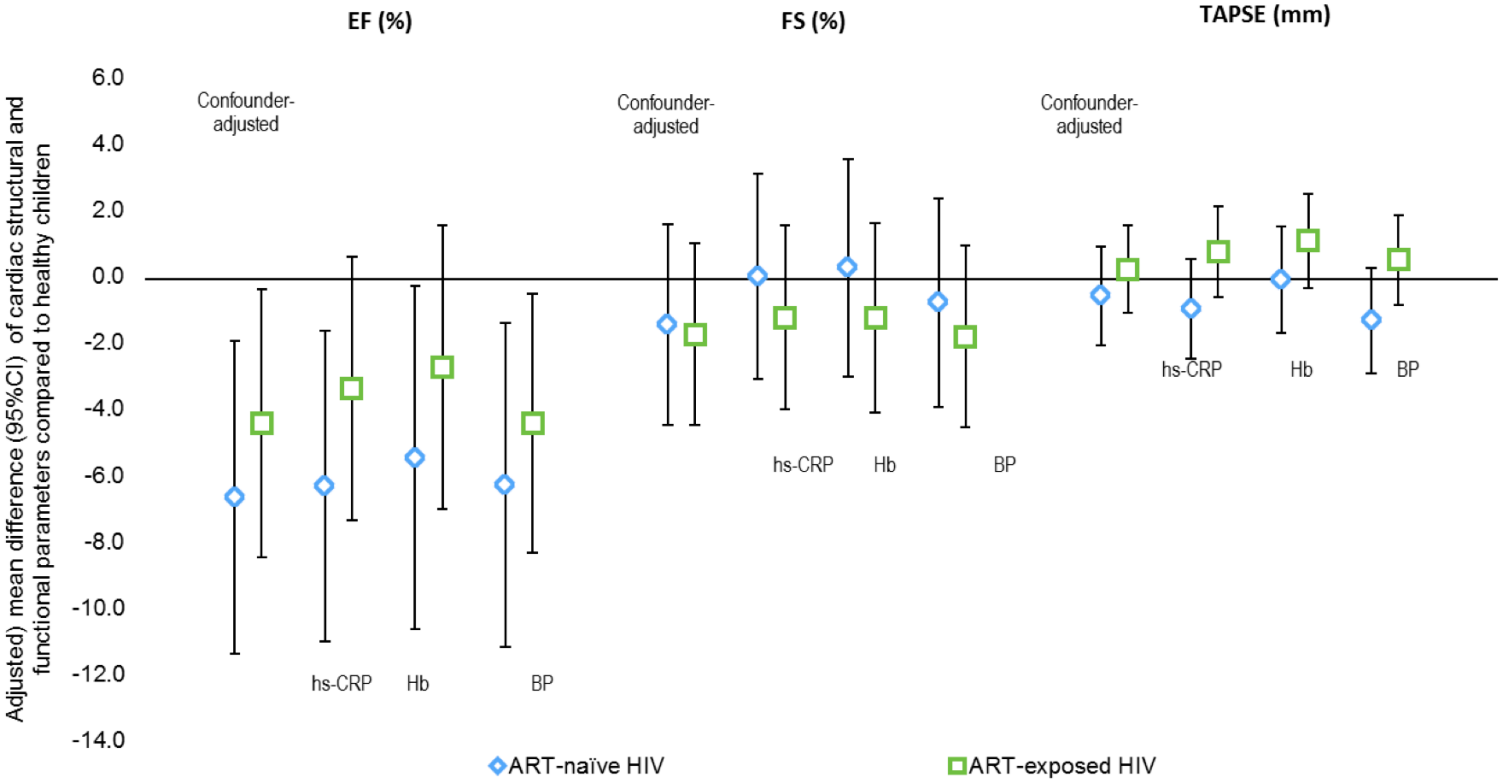
Influence of potential explanatory variables on cardiac functional estimates in ART-naive and ART-exposed HIV infected children. Note: EF = ejection fraction; FS = fractional shortening; TAPSE = tricuspid annular plane systolic excursion; Hb = hemoglobin level; BP = systolic and diastolic blood pressure.

In terms of left ventricular systolic function (Table 3), ART-naïve HIV infected children had lower LVEF than healthy children (adjusted difference -6.7%, p = 0.006); ART-exposed HIV infected children showed this association to a lesser degree (adjusted difference -4.5%, p = 0.03). ART-naïve children tended to have lower FS (-2.5%, p = 0.06) and poorer RV systolic function as reflected in lower TAPSE (-3.6mm, p<0.001), but not after adjustment for age and height (-1.3%, p = 0.40 and -0.6mm, p = 0.42 for FS and TAPSE, respectively). Degree of inflammation seemed to explain reduced LVEF in ART-exposed HIV-infected children, but not in the ART-naïve. In contrast, hemoglobin level explained decreased systolic function in both ART-naïve and, to a larger extent, the ART-exposed HIV infected children (Fig 2).

**Table 3:** Effects of HIV infection on cardiac functions.

		Crude				Model 1			Model 2		
			Mean difference	95%CI	P	Mean difference	95%CI	P	Mean difference	95%CI	P
LVEF (%)	Non-HIV		Ref			Ref			Ref		
	ART-naïve HIV	All 50	-6.42	-10.35 to -2.49	0.002	-7.07	-11.33 to -2.81	0.001	-6.68	-11.41 to -1.95	0.006
		41 Never ART	-6.10	-10.00 to -2.12	0.002	-6.86	-11.16 to -2.55	0.002	-6.18	-10.97 to -1.40	0.01
	ART-exposed HIV		-4.66	-8.41 to -0.90	0.02	-4.92	-8.74 to -1.10	0.01	-4.46	-8.49 to -0.42	0.03
LVFS (%)	Non-HIV		Ref			Ref			Ref		
	ART-naïve HIV	All 50	-2.54	-5.21 to 0.13	0.06	-2.14	-4.95 to 0.67	0.13	-1.30	-4.32 to 1.72	0.40
		41 Never ART	-3.14	-5.88 to -0.40	0.03	-2.98	-5.95 to -0.01	0.05	-2.33	-5.55 to 0.88	0.15
	ART-exposed HIV		-2.47	-5.09 to 0.16	0.07	-2.27	-4.93 to 0.39	0.10	-1.78	-4.51 to 0.95	0.20
TAPSE (mm)	Non-HIV		Ref			Ref			Ref		
	ART-naïve HIV	All 50	-3.64	-5.15 to -2.12	<0.001	-2.20	-3.63 to -0.77	0.003	-0.61	-2.17 to 0.79	0.42
		41 Never ART	-3.64	-5.22 to -2.05	<0.001	-1.77	-3.29 to -0.25	0.02	0.20	-1.36 to 1.75	0.80
	ART-exposed HIV		-1.26	-2.73 to 0.22	0.10	-0.71	-2.04 to 0.63	0.30	0.20	-1.11 to 1.53	0.77

Note: Model 1: adjusted for age; Model 2: adjusted for age and height

Analyses excluding ART-naïve HIV infected children who had a brief period of treatment in the past (Tables 2 and 3) or 2 ART-exposed children with potential previous compliance issue (data not shown) revealed similar findings.

## Discussion

This study shows that ART-naive HIV infected children had markedly lower LV ejection fraction with chamber dilation than healthy children. ART-exposed HIV infection showed structural changes with thicker LV posterior wall, chamber dilation, and higher LV mass, and a lesser degree of decreased systolic function. Degree of inflammation seemed to be involved in the effects of ART-exposed HIV infection on both structural and functional changes, except for the higher LV mass, while for the ART-naïve, it partially explained the larger LV internal diameter, but provided little explanation for the decreased systolic function. Lower hemoglobin level appeared to account for larger internal diameter in both ART-naïve and ART-exposed and decreased systolic function particularly in ART-exposed HIV infected children.

Previous studies evaluating cardiac involvement in HIV infection in children involved larger sample size [5] than ours or used more refined techniques, such as strain analysis.[6] However, those studies, whether in the pre- or post- highly active—ART era, typically looked at either naive [6] or ART exposed populations.[3] and ones including both, [13] did not clearly delineate between ART-naive or ART-exposed HIV infection. We had the unique opportunity to study the cardiac status in both ART-naïve and ART-exposed HIV infected children, within the same time frame in the post highly active ART era rather than making a historical comparison. In Indonesia, universal HIV screening in pregnancy is unfortunately not widely applied, resulting in inadequate prevention of mother-to-child transmission and late identification of new pediatric cases. Our design and analytical approach disentangles the effects of ART-naïve from ART-exposed HIV infection. Our use of dedicated healthy children as a reference, rather than published normal values, [14] hospital records of normal echocardiograms, [6] or HIV-exposed uninfected children [13] is a particular strength. With patients referred for echocardiography who showed no cardiac anomalies [6] as reference, the indication for echocardiography may still bias findings. Although some previous studies applied population matching or restrictions, the mandatory corresponding conditional analyses were not applied, and to our understanding none were appropriately adjusted for confounding. Finally, we explored several possible explanatory analyses, which to our knowledge has never been done previously.

We only addressed basic parameters of cardiac structures and functions in HIV-infected children and had not yet looked at the occurrence of cardiac disease entities, which are probably more relevant for pediatric clinical practice.[5] However, our approach may serve as a starting point in analyzing fundamental changes in cardiac structures and functions preceding the occurrence of such entities. This involves consideration of certain slightly different definitions and cut offs, such as different criteria to define cardiomyopathy which may not have direct prognostic relevance.

We found that ART-naïve HIV infected children had compromised ventricular systolic function with chamber dilatation, while the ART-exposed children had less functional impairment despite thicker LV posterior wall, larger internal diameter, and higher mass. This was never explicitly reported before as in most studies, ART-naïve and ART-exposed HIV-infected children were pooled into one group, showing various cardiac structural changes, such as hypertrophic or abnormal ventricular mass, [15] chamber dilatation, as well as systolic or diastolic dysfunctions.[5] Our findings imply that ART, either by controlling viral replication or exerting direct cardio protective effects, may improve cardiac functions as was shown by following up HIV-infected children who switched from no or single ART to highly active three drug ART regimens [4] and a cross-sectional study demonstrating comparable LV systolic function of treated HIV-infected to healthy adolescents.[16] Thicker ventricular wall, chamber dilatation, and higher mass in ART-exposed HIV infected children may reflect treatment-induced compensatory mechanisms to bear the extra load, [17] by which the systolic function was relatively preserved or, on the other hand, effects of the interaction between treatment and residual HIV infection that may be disadvantageous in the long term, necessitating further follow up. Previous findings about LV mass and dimensions were inconclusive. Some studies suggested that ART, either given for prevention in fetal/early postnatal life of HIV-exposed uninfected infants or as a treatment, was associated with reduced LV mass and septal thickness [18], [19], while later findings showed no differences in left ventricular dimensions compared to HIV-unexposed reference group although analysis restricted to HIV-exposed uninfected children revealed that ART exposure in the first trimester of pregnancy resulted in thicker posterior wall.[20] Furthermore, earlier findings, including an autopsy study, showed a progressive increase of LV mass and end-diastolic dimension in mostly severely symptomatic children who were in majority (60–90%) treated, either by zidovudine only or combination therapy.[21] These differences may occur because of different study populations, comparisons, and analytical approaches, including the use of adjusted measures for nutritional status, which in our view may have a strong association with both HIV infection status and cardiac structures and which we therefore accounted for. We infer that higher LV mass, chamber dilatation, and thicker ventricular wall among our ART- HIV-infected subjects results from the interaction between highly-active ART and partially controlled HIV infection, as around 60% of them were still in a severe-to-advanced clinical stage.

Mechanisms underlying cardiac involvement in HIV-infected children remain poorly understood. Multiple factors may be involved, including HIV-induced cytokine or proteolytic enzyme release, co-infection, nutritional deficiency, autoimmune response to infecting pathogens, ART cardio toxicity, or direct viral cell invasion.[22] Our findings suggest that HIV infection itself, by whatever mechanisms, first cause functional cardiac changes by compromised systolic function. Subsequent treatment, probably by controlling HIV replication, seems to partially restore cardiac function, which might be at the expense of inducing structural abnormality. The suboptimal LVEF that was still observed in our ART-treated HIV infection subjects may indicate residual damage due to previously untreated infection. Previous studies investigating cardiac involvement in HIV-infected subjects rarely, if at all, explored potential mechanisms underlying the reported associations. Based on our findings, degree of inflammation may mediate the effects of ART-exposed HIV infection on thicker posterior wall and slightly reduced LVEF, but provides less explanation for the effects of ART-naive HIV infection on decreased LVEF. On the other hand, lower hemoglobin seemed to account for chamber dilation in both groups and lower LVEF, particularly in ART-exposed HIV infected children. Although family history of CVD was more frequent in our HIV-infected subjects, we believe that it reflects a clustering of high risk behaviors, such as smoking, rather than a causal relation with the observed cardiac structural or functional changes.

From the perspective of clinical practice, our findings underline the detrimental cardiac effects of ART-naïve HIV infection, which should raise awareness to provide early treatment and more elaborate care for this kind of patients. As ART does not completely solve the cardiac problems and even may have long term disadvantageous effects, a regular follow up of cardiac status may be required for HIV-infected children. This is of particular importance as increased wall thickness and depressed systolic function have been reported as a significant predictor for mortality in HIV-infected children independent of CD4 cell count.[23]

In conclusion, ART-naive HIV infected children have a substantial decrease in cardiac systolic functions accompanied by chamber dilatation. In ART-exposed HIV infected children, thicker ventricular walls with higher mass and chamber dilatation dominate, with less functional impairment.

## Supporting Information

S1 Dataset Minimum dataset file. (SAV)
